# Redesigning Arenicin-1, an Antimicrobial Peptide from the Marine Polychaeta *Arenicola marina*, by Strand Rearrangement or Branching, Substitution of Specific Residues, and Backbone Linearization or Cyclization

**DOI:** 10.3390/md17060376

**Published:** 2019-06-23

**Authors:** Dmitriy S. Orlov, Olga V. Shamova, Igor E. Eliseev, Maria S. Zharkova, Oleg B. Chakchir, Nikolinka Antcheva, Sotir Zachariev, Pavel V. Panteleev, Vladimir N. Kokryakov, Tatiana V. Ovchinnikova, Alessandro Tossi

**Affiliations:** 1 Institute of Experimental Medicine, 12 Academic Pavlov str., St. Petersburg 197376, Russia; ds-orlov@yandex.ru (D.S.O.); manyvel@mail.ru (M.S.Z.); kokryak@yandex.ru (V.N.K.); 2 St. Petersburg Academic University, 8/3 Khlopina str., St. Petersburg 194021, Russia; eliseev@spbau.ru (I.E.E.); chakchir@spbau.ru (O.B.C.); 3 Department of Life Sciences, University of Trieste, Building Q, Via Giorgieri 5, 34127 Trieste, Italy; nantcheva@gmail.com (N.A.); atossi@units.it (A.T.); 4 International Centre for Genetic Engineering and Biotechnology, AREA Science Park, Padriciano 99, 34149 Trieste, Italy; sotir@icgeb.org; 5 M.M. Shemyakin & Yu.A. Ovchinnikov Institute of Bioorganic Chemistry, the Russian Academy of Sciences, Mikhluho-Maklaya str. 16/10, Moscow 117997, Russia; alarm14@gmail.com (P.V.P.); ovch@ibch.ru (T.V.O.); 6 Department of Biotechnology, I.M. Sechenov First Moscow State Medical University, Moscow 119991, Russia

**Keywords:** marine peptides, arenicin-1, molecular symmetry, structure–activity relationship, antibacterial, cytotoxic, chemical synthesis, molecular dynamics

## Abstract

Arenicin-1, a β-sheet antimicrobial peptide isolated from the marine polychaeta *Arenicola marina* coelomocytes, has a potent, broad-spectrum microbicidal activity and also shows significant toxicity towards mammalian cells. Several variants were rationally designed to elucidate the role of structural features such as cyclization, a certain symmetry of the residue arrangement, or the presence of specific residues in the sequence, in its membranolytic activity and the consequent effect on microbicidal efficacy and toxicity. The effect of variations on the structure was probed using molecular dynamics simulations, which indicated a significant stability of the β-hairpin scaffold and showed that modifying residue symmetry and β-strand arrangement affected both the twist and the kink present in the native structure. In vitro assays against a panel of Gram-negative and Gram-positive bacteria, including drug-resistant clinical isolates, showed that inversion of the residue arrangement improved the activity against Gram-negative strains but decreased it towards Gram-positive ones. Variants with increased symmetry were somewhat less active, whereas both backbone-cyclized and linear versions of the peptides, as well as variants with R→K and W→F replacement, showed antimicrobial activity comparable with that of the native peptide. All these variants permeabilized both the outer and the inner membranes of *Escherichia coli*, suggesting that a membranolytic mechanism of action was maintained. Our results indicate that the arenicin scaffold can support a considerable degree of variation while maintaining useful biological properties and can thus serve as a template for the elaboration of novel anti-infective agents.

## 1. Introduction

The growing resistance of pathogenic bacteria to currently used drugs dictates an urgent search for novel antibiotics. The antimicrobial peptides produced by the innate immune system of animals or plants are considered as potential new agents to combat drug-resistant bacteria, since they have a potent and rapid antimicrobial action and act via a multi-target mode of action. The innate immune system plays a vital role in the host defense of invertebrates, in particular, as their adaptive immunity is poorly developed, so that they have evolved a wide range of antimicrobial peptides (AMPs). Those of marine invertebrates can be considered as one of the most promising sources of new and effective antibiotics, especially AMPs from species inhabiting coastal zones, which are environments teeming with microbes, where the peptides are vital to help them avoid infection.

The subject of our study is a cationic antimicrobial peptide, arenicin-1, from the coelomocytes of one such invertebrate animal—the lugworm *Arenicola marina* [1]. This peptide adopts a β-hairpin structure [2,3,4] and possesses a potent microbicidal activity towards a broad spectrum of Gram-negative and Gram-positive bacteria (including drug-resistant clinical isolates), as well as towards fungi. However, it also displays a substantial cytotoxicity towards mammalian cells [1,2,3]. The mechanism of antimicrobial action of arenicin-1 is associated with a distinctive membranolytic activity [5,6,7,8,9,10,11,12]. Its potent antibiotic activity makes this peptide a fascinating lead for designing variants to test which features determine the antimicrobial and/or cytotoxic capacities and to disclose if they can be in some way extricated.

**Figure 1:**
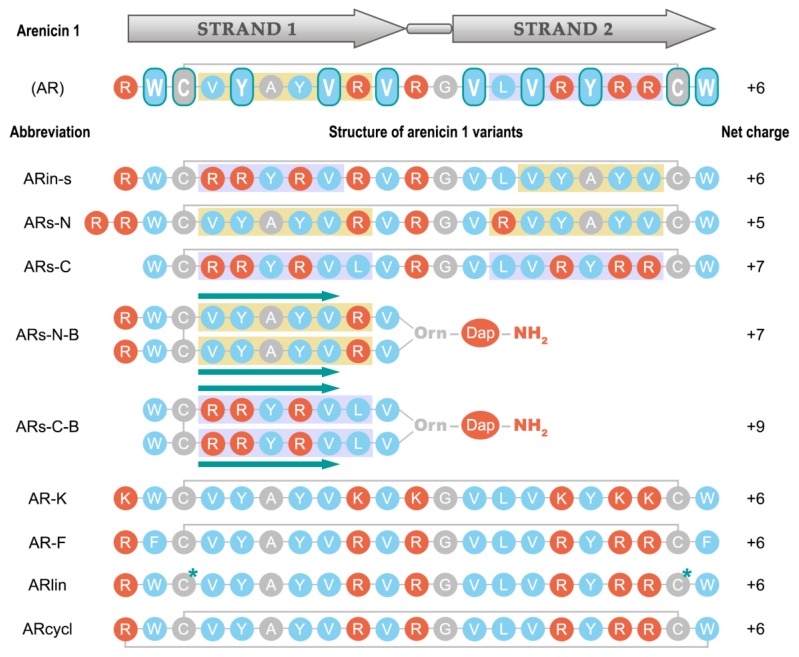
Pseudosymmetric sequence of the arenicin-1 molecule and structure of its designed analogues. The hydrophobic residues are marked in blue, the polar residues are in red, and the palindromic arrangement of the WCxxYxxVxVxxVxVxYxxCW motif in the initial arenicin-1 sequence is indicated with rounded boxes. The VYYAYV(R) and (L)VRYRR motifs present in strands 1 and 2 of arenecin-1 are, respectively, highlighted by yellow and blue-grey boxes and were swapped (ARin-s), placed symmetrically with inversion (ARs-N and -C), or branched from a central residue (ARsin-N-B and ARs-C-B) in variants designed to test the role of the residue symmetry. Asterisks (*) in the linear version of arenicin-1 (ARlin) mark the sites of Cys alkylation with iodoacetamide. Green arrows accentuate the parallel arrangement of the strands in ARs-N-B and ARs-C-B peptides, obtained by linking the “branches” of the hairpin to the α and δ amines of an ornithine residue (Orn), in turn coupled to an amidated diaminopropionic acid (Dap) group at the C-terminus (Figure 7).

Though some of the structural characteristics of arenicins have already been explored, the significance of an intriguing feature of this molecule—its pseudosymmetric residue arrangement—remains unexplored (Figure 1).

The β-hairpin structures of arenicins show marked right-handed twist and kink in aqueous solution, as revealed by NMR spectroscopy [2,4] as well as by molecular dynamics simulation [6]. These features effectively allow the side chains of hydrophobic residues to be screened in an aqueous environment, thus reducing the overall amphiphilicity of the peptide. In more anisotropic, membrane-mimicking environments, arenicins form dimers stabilized by hydrogen bonds between parallel N-terminal β-strands in two neighboring molecules [5]. Dimerization occurs at the membrane surface and induces a substantial conformational change, so that the molecules adopt almost planar amphipathic β-sheet structures [8,13]. This more regular conformation appears conducive to subsequent oligomerization and membrane disruption and therefore appears to be a relevant feature for antibacterial activity. Conformational regularity might be favored by an increased symmetry in residue arrangement.

The present study aimed to probe for a possible biological significance of the apparent residue symmetry displayed by arenicin-1 molecules and to determine whether altering or increasing this symmetry could result in improved antimicrobial characteristics, also in view of their potential as leads for novel anti-infective agents. We furthermore wanted to test how an increased rigidity of the β-hairpin could affect the biological properties as well as the roles played by specific residues.

Several variants of arenicin-1 were therefore elaborated for a structure–activity relationship study to analyze the role of sequence symmetry, residue content, and conformational rigidity in microbicidal efficacy, membranolytic activity, and cytotoxicity towards mammalian cells. We constructed a set of peptides with a varying degree of symmetrization of the amino acid sequence and speculated that this could result in a more planar arrangement of the peptide hairpin, with a reduction of the kink and twist observed in the strands of the native peptide’s structure. To test this, we performed extensive molecular dynamics simulations of the peptides, using different initial conformations, and analyzed their folding capacity, stability, secondary structural features such as kink and twist angles, and hydrogen bonding patterns.

Additional variants were also studied, in which key residues, such as the arginines that provide the peptide’s charge or the tryptophan residues that flank the β-hairpin, were respectively replaced with lysine and phenylalanine. We furthermore prepared a linear variant of arenicin-1 with reduced and alkylated (iodoacetamidated) cysteine residues to probe the significance of the β-hairpin scaffold and, finally, a backbone-cyclized arenicin-1 analogue to explore if the increased rigidity that is likely to result from this type of cyclization could affect activity and/or toxicity towards mammalian cells.

## 2. Results

Arenicin-1 (AR) adopts a characteristic β-hairpin structure stabilized by the presence of a disulfide bridge and displays a peculiar symmetry in residue arrangement along the two strands (Figure 1). It is a fascinating scaffold for designing variants to test features that may affect the molecule’s antimicrobial and/or cytotoxic capacities.

### 2.1. AR Variants Design

ARin-s was designed to alter the sequence symmetry by inverting the VYAYV and VRYRR motifs, respectively present in the N- and C-terminal strands of wild-type AR (Figure 1). ARs-N had increased symmetry based on only the VYAYVR motif of the N-terminal strand; an additional Arg residue was added to offset the decrease in overall charge that this entails. ARs-C, instead, had increased symmetry based on the VRYRR motif of the C-terminal strand. To increase symmetry even further, two non-proteinogenic variants—ARs-N-B and ARs-C-B—were constructed by branching either the VYAYVRV sequence of strand 1 or the VLVRYRR sequence of strand 2 (fragments 1–10 and 13–21, respectively, Figure 1) from the α and δ amines of an ornithine (Orn) residue. This resulted in a parallel arrangement of the hairpin strands, so that the obtained peptides had two N-termini. The branching Orn residue was linked to an amidated diaminopropionic acid (Dap) residue at the peptide’s C-terminus, in an attempt to maintain a structure of the branching site as far as possible isosteric with the Arg residue normally present in the turn region (see Figure 7 in the Methods section).

We also constructed a backbone-cyclized version of AR (ARcycl) by linking the N-terminus to the C-terminus using an innovative native chemical ligation (NCL) protocol. It has been reported that the introduction of a second disulfide bridge in arenicin increases its rigidity and that this has beneficial effects on both antimicrobial activity and toxicity [14]. However, this required replacing internal residues (namely, Val^8^ and Val^13^) with Cys, while backbone cyclization of the N- and C-termini, that are spatially close in the peptide β-hairpin, should be less invasive.

Additional variants were prepared with substitution of key residues. In AR-K, all Arg residues were substituted with Lys to probe the relevance of these charged residues; analogously, in the AR-F variant, Trp was replaced with Phe to probe the relevance of the flanking tryptophan residues. Finally, we also prepared a linear variant of arenicin-1 with reduced and carboxamidomethylated (iodoacetamidated) cysteine residues (RCM-Cys).

### 2.2. Assessing the Structure Using Molecular Dynamics Simulations

In our computational analysis of arenicin-1 analogues, we first developed a quantitative measure of sequence symmetry by aligning the original and the inverted primary structures. As can be appreciated from their sequences (Figure 1), the “branched” peptides ARs-N-B and ARs-C-B are ideal palindromes, having not only identical sequences of both strands but also the same orientation.

We then performed an extensive molecular dynamics (MD) simulation of the peptides in an aqueous environment to analyze their structure and dynamics. The peptides were modelled with different starting conformations, in the presence or absence of the disulfide bonds. The linearized version of the original arenicin-1, as well as the variant with reduced, carboxamidomethylated cysteines (ARlin) and the linearized versions of the ARin-s and ARs-C variants, all demonstrated the ability to fold rapidly from a fully extended linear conformation to a well-defined β-hairpin structure, which was relatively stable even without the disulfide bond. The process of peptide folding was monitored by measuring both the distance from the initial structure and the appearance of secondary structure elements. The folding of ARs-C, with rapid emergence of the characteristic β-strands connected by a turn, is shown in Figure S1. ARin-s and ARs-C were also modelled from more natural β-hairpin starting structures, obtained using replica-exchange Monte Carlo simulations in the *Quark* program, which also confirmed a high stability of this conformation even in the absence of the disulfide bond.

**Figure 2:**
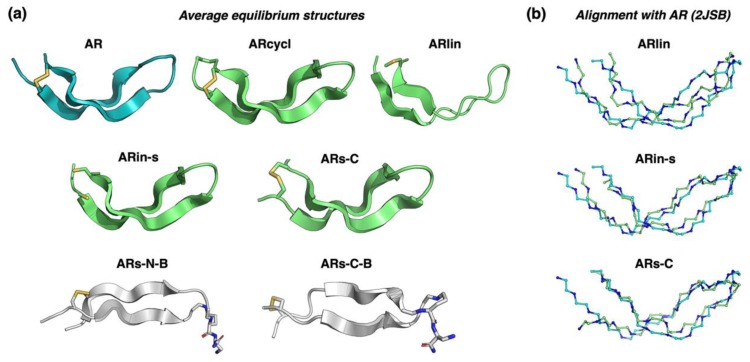
Average equilibrium structures obtained for all studied peptides by molecular dynamics (MD) simulations. (**a**) Schematic ribbon representation, parallel and antiparallel β-strands are based on secondary structure assignment by the STRIDE algorithm. (**b**) Structural alignment of ARlin, ARin-s, and Ars-C with experimental arenicin-1 structure 2JSB. Only backbone atoms are shown, the coloring matches panel (**a**).

The ARs-N-B and ARs-C-B variants, instead, failed to fold from an initial extended linear conformation, in the timescales used for our simulations. Therefore, we used a manually constructed β-sheet structure with a disulfide bond to perform simulated annealing experiments. These resulted in the formation of relatively flat β-sheet structures, which were further equilibrated by an additional MD simulation. The resulting average structures obtained for all modelled peptides are shown in Figure 2a, in which it can be clearly seen that the β-hairpin structures of ARin-s and ARs-C show the same characteristic kink as AR, while the parallel β-hairpin of ARs-N-B and ARs-C-B are flatter and less twisted.

The increase in sequence symmetry in the parallel β-stranded branched peptides made their planar spatial structures the most distant from that of the original arenicin-1. Data on the structural distance from the experimental arenicin-1 structure (root-mean-square deviation, RMSD), secondary structure content, and average number of mainchain hydrogen bonds for each peptide are provided in Table 1. These show that the linearized version with reduced, carboxyamidomethylated cysteines (ARlin) formed a more distorted β-hairpin, with lower secondary structure content and greater distance from the experimental structure than the other antiparallel β-hairpin structures. On the other hand, the cyclic variant (ARcycl) and ARin-s were almost indistinguishable from the original arenicin-1, as shown in the structural alignments in Figure 2b, so that the rearrangement of residues in the two strands or backbone cyclization did not seem to greatly affect the overall conformation. It is interesting to note that the backbone cyclization can accommodate quite a tight cycle based on only five residues (CWRWC), even though the three central residues are bulky, so that the motif could be quite rigid.

**Figure 3:**
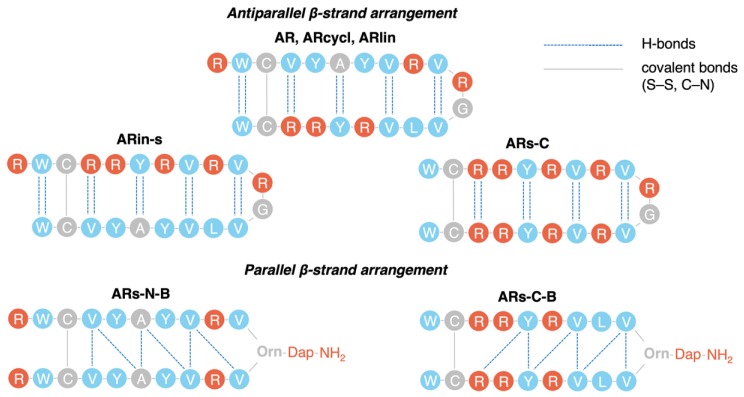
Different hydrogen bonding patterns observed for arenicin variants. AR, ARin-s, and ARs-C show the same H-bonding pattern (dotted blue lines) typical of antiparallel β-strands (two H-bonds connect the α-amine and α-carboxyl groups of an amino acid on one side of the hairpin with the α-amine and α-carboxyl groups of an amino acid on the other side of the hairpin). ARs-N-B and ARs-C-B instead show the pattern typical of parallel β-strands (two H-bonds connect the α-amine and α-carboxyl groups of an amino acid on one side of the hairpin with the α-amine and α-carboxyl groups of two different amino acids on the other side). The disulfide bond is indicated by a solid grey line, as well as branching from the ornithine residue α- and δ-amines (Figure 7).

The parallel, branched β-hairpin peptides ARs-N-B and ARs-C-B were structurally the most distant from the original conformation, with RMSD > 4 Å for ARs-N-B. Moreover, although the average number of intramolecular main-chain hydrogen bonds was similar for all peptides, AR, ARin-s, ARs-C displayed the standard hydrogen bonding pattern of an antiparallel β-sheet (Figure 3), while ARs-N-B and ARs-C-B showed a bonding pattern characteristic of a parallel β-sheet. Supposedly, this difference in intramolecular hydrogen bonding could affect also intermolecular H-bonding and so alter the mechanism of dimerization and oligomerization on the membrane surface. 

The structural stability of the peptides was analyzed by calculating the fluctuations (root-mean-square fluctuations, RMSF) of the backbone C^α^ atoms at equilibrium. All peptides demonstrated a fluctuation pattern typical of a β-sheet with a higher mobility at the termini and near the turn connecting the two β-strands (Figure S2a). The peptide ARs-C-B exhibited a remarkably high structural stability, with low fluctuations even on the β-strand termini. This behavior could be attributed to an additional stabilization due to stacking of two N-terminal tryptophan residues, which is shown in Figure S2b.

**Figure 4:**
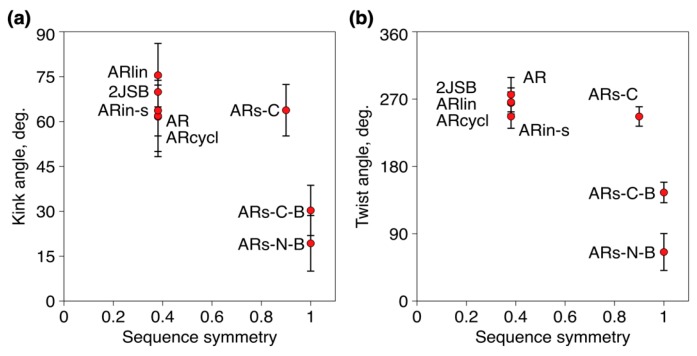
Average kink (**a**) and twist (**b**) angles for the peptides as a function of their sequence symmetry. Sequence symmetrization in ARs-N-B and ARs-C-B results in significantly lower kink and twist.

Finally, we analyzed the effect of sequence symmetry on the overall geometry of the peptide. For each peptide, we determined the average twist and kink angles from the equilibrium parts of the MD trajectories. We then plotted the twist and kink angles versus sequence symmetry for each peptide, as shown in Figure 4. As expected, and in agreement with the MD simulations, the more symmetrical sequences of ARs-N-B and ARs-C-B resulted in significantly lower twist and kink of the peptides, which both had nearly planar parallel β-hairpin structures, quite distant from the original arenicin-1 structure and rather uncommon for β-hairpin peptides, which cannot by, definition, have symmetric, parallel β-strands.

### 2.3. Antimicrobial Activity

We tested the antimicrobial activity of the peptides against a set of Gram-negative and Gram-positive bacteria, including drug-resistant clinical isolates. As shown using the broth microdilution assay (Table 2), the peptide with inverted symmetry (ARin-s) had a reduced activity against Gram-negative strains and lost activity against Gram-positive ones. This may be due to the replacement of the VYAYV motif in the N-terminal strand, which is reported to be important for oligomerization, with the inverted C-terminal motif RRYRVL. However, the fact that the variant with a symmetrically placed RRYRVL motif in both strands (ARs-C) maintained a substantial activity, while the one with a symmetrically placed VYAYV motif lost activity, suggests that this type of oligomerization is only one of the factors determining activity and that the C-terminal strand may also have a significant role. ARs-C (+7) may act as a monomer for which the higher charge is beneficial, and while we attempted to maintain a reasonable cationicity (+5) for ARs-N by adding an Arg residue at the N-terminus, it was still reduced with respect to arenicin-1 (+6). It is interesting that the same trend was observed for the “branched” variants, where ARs-C-B (+9), based on the RRYRVL motif of the C-terminal strand, was more active than ARs-N-B (+7), based on the VYAYV motif of the N-terminal strand, but the latter becomes significantly more active than the less cationic ARs-N peptide based on a canonical antiparallel β-hairpin.

The backbone-cyclized and linear versions of arenicin-1, as well as the peptides with Arg replaced with Lys (AR-K) and Trp replaced with Phe (AR-F), showed an antimicrobial activity comparable with that of the native peptide. However, it was not possible to determine from the minimal inhibitory concentrations (MIC) values alone if and how the mechanism of action had changed. One may simply suggest that it continued to involve an initial interaction with the membrane determined by electrostatic interactions and that the subsequent insertion into the membrane was due to the presence of hydrophobic residues, but the mechanism of the subsequent membrane permeabilization (which involves peptide oligomerization) could be affected. Our MD simulations would in any case suggest that the β-hairpin structure was maintained in all these variants, even the linear one, with a strong propensity for the scaffold to fold into this conformation. It is interesting to note that, while altering the symmetry reduced the antimicrobial activity against drug-resistant clinical isolates in particular, backbone cyclization of arenicin-1 resulted in improved activity towards these isolates.

To obtain a global assessment of the effect on antimicrobial activity, the geometric mean of the MIC (G-MIC) was calculated, and from this, the overall improvement or impoverishment with respect to arenicin-1. In general, inverting the symmetry or decreasing the charge had detrimental effects. Turning the peptide into a parallel β-hairpin also did not improve activity. Linearizing the peptide did not seem to significantly affect its activity, while substituting Lys for Arg and Phe for Trp seemed to slightly improve it. Taken together, these results suggest that these modifications did not markedly improve the already potent antibacterial activity of the parent peptide but rather subtly alter its activity spectrum.

### 2.4. Effect of Arenicin Variants on Bacterial Membrane Integrity

**Figure 5:**
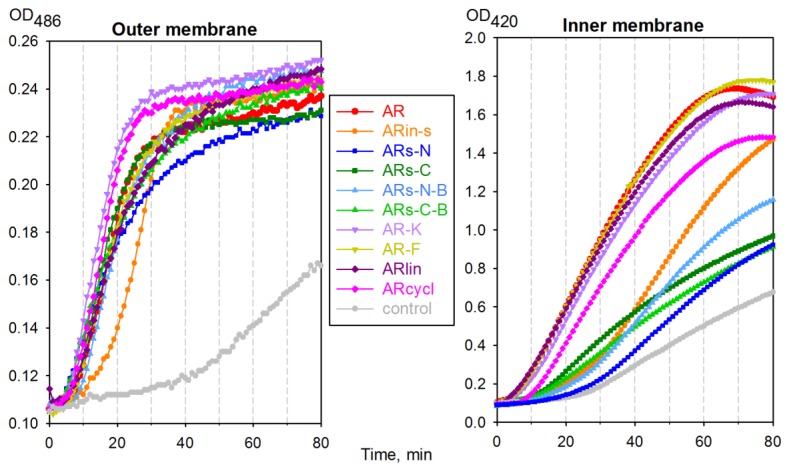
Permeabilizing effect of arenicin-1 variants on *E. coli* ML35p outer and cytoplasmic membranes. The incubation wells contained 10mM sodium phosphate buffer, 100 mM NaCl, 2.5 × 10^7^ colony-forming units (CFU) of washed, stationary-phase *E. coli* ML35p, and the peptides of interest at a concentration equivalent to 2 × MIC, or an equivalent volume of acidified water (controls).

The *Escherichia coli* ML35p strain expresses a plasmid-encoded periplasmic β-lactamase and is constitutive for cytoplasmic β-galactosidase, while lacking lactose permease [15]. This makes it very useful to monitor the permeabilization of both its outer and inner membranes, using real-time assays. From Figure 5, it can be seen that the outer membrane became fully permeable to nitrocefin, a β-lactamase substrate, ~30 min after adding most arenicin-1 analogs. However, all variants with a modified symmetry demonstrated a decreased ability to permeabilize the inner membrane of *E. coli* ML35p, suggesting a reduced membranolytic activity.

It is interesting to note that, whereas some variants did show an improved capacity to permeabilize the outer membrane with respect to arenicin-1 (e.g., AR-K and ARcycl), none of the variants (with the possible exception of AR-F) showed an improved capacity to permeabilize the cytoplasmic membrane.

### 2.5. Cytotoxicity of Arenicin-1 Variants towards Mammalian Cells

The toxicity of arenicin peptide variants towards eukaryotic cells was tested using the MTT assay on the human erythroleukemia cell line (K-562). The arenicin variants with Arg-to-Lys (AR-K) and Trp-to-Phe (AR-F) substitutions, which exerted a slightly improved antimicrobial activity compared to the native peptide (see Table 2), were also found to be somewhat more cytotoxic, the half-maximal inhibitory concentration (IC_50_) decreasing by a factor of 2 (Table 3). This is quite typical of membranolytic AMPs, for which a higher antibacterial activity is often accompanied by an increased toxicity to host cells [16,17].

The cytotoxic activity of the backbone-cyclized variant ARcycl and of variants modified to increase the symmetry in an antiparallel β-sheet structure (ARs-N and ARs-C) was comparable (ARcycl, ARs-N) or only moderately increased (ARs-C), with respect to that of the original peptide. On the other hand, both of the “branched” symmetrical variants (ARs-N-B and ARs-C-B) and the linearized arenicin (ARlin) showed at least a two-fold reduction of cytotoxicity. The same was observed for ARin-s with inverted symmetry, but in this case, it was accompanied by a significantly lower antibacterial activity.

**Figure 6:**
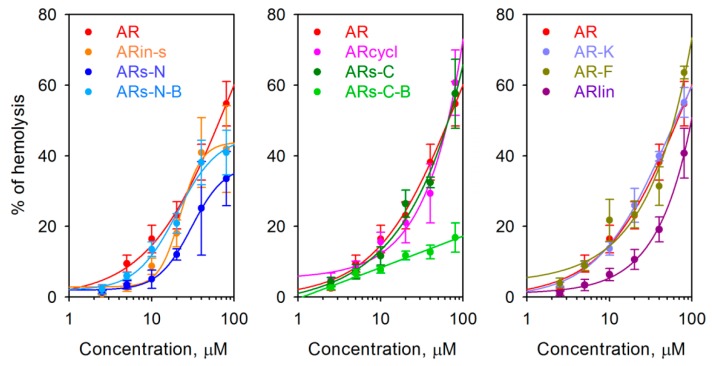
The hemolytic activity of arenicin-1 variants towards human red blood cells (RBC). All values are means ± SD and were derived from 3–8 experiments which were performed in triplicates.

The impact of the peptides on eukaryotic membranes was also analyzed using the hemolysis assay, with peptide concentrations up to 80 µM (Figure 6). None of the modifications completely abolished the relatively high hemolytic activity of wild-type arenicin, although the branched variant based on the C-terminal motif (ARs-C-B), which was moderately active on bacteria, demonstrated a significantly lowered ability to lyse the erythrocytes. On the other hand, other variants with improved antimicrobial activity (e.g., AR-F and AR-K) did not show a significantly increased hemolytic activity, based on the HC_50_ value.

We used both the MTT and hemolysis data to make an in vitro estimate of selectivity indices (SI) of the peptides (see Table 3), as these help characterize the width of the ‘therapeutic window’ of a compound and thus its suitability for possible therapeutic application. We used the G-MIC as a measure of the overall antimicrobial activity of the peptides (see Table 2) and the half-maximal MTT inhibitory concentration (IC_50_) or half-maximal hemolytic concentration (HC_50_) as a measure of their toxicity. In the case of hemolytic activity, only 5 of the 10 investigated variants reached their HC_50_ at a concentration of 80 μM, which somewhat limited the SI assessment. For a more precise comparison of the SI, we therefore calculated the HC_15_, a hemolysis level which could be reliably calculated for all tested peptides on the basis of the experimental data (Figure 6). Both hemolysis- and MTT-derived selectivity index estimates (SI_1_ and SI_3_, see Table 3) indicated that the branched symmetrical arenicin-1 variant based on the C-terminal motif (ARs-C-B) and the linearized arenicin (ARlin) possessed the best combination of antibacterial and cytotoxic properties, amongst the tested arenicin-1 analogues. ARs-C-B showed over a three-fold improvement in SI_3_ compared with native arenicin. The variants with the poorest SI characteristics were the one with inversed symmetry (ARin-s: low antimicrobial activity but relatively high cytotoxicity) and the N-terminal motif-based symmetrical arenicin ARs-N.

## 3. Discussion

Marine animals are recognized as a rich source of potentially useful novel bioactive substances, including new anti-infective drugs. Several AMPs from marine invertebrates have been described to date, such as the penaeidins from shrimp [18], mytilins from mussels [19], aurelin from jellyfish [20], tachyplesins from the horse-shoe crab [21], arasin from the spider crab [22], and several others. One of the most potent AMPs is arenicin-1, from the lugworm *A. marina* [1]. However, to be suitable as a lead compound for drug development, this highly active peptide requires optimization, as it is also significantly hemolytic.

We observed that this peptide possesses a rather unique pseudosymmetric structure and explored the significance of this feature with respect to its antimicrobial activity as well as its cytotoxicity for mammalian cells. This peptide presents two Trp residues at each terminus and three Tyr residues distributed along the β-strands. A study of other tryptophan-rich peptides, such as the bovine, cathelicidin-derived peptides tritrpticin and indolicidin, has shown that sequence symmetrization can result in improvement of the antimicrobial activity, accompanied by a decreased hemolytic action [23]. A zipper-like symmetric arrangement of aromatic residues along the strands can also increase the stability of β-hairpin AMPs, increasing their activity [24]. Furthermore, synthetic, disulfide-stabilized, β-hairpin peptides based on symmetric sequences of a VR motif also provide an appreciable, broad-spectrum antimicrobial activity with a membranolytic mechanism [25].

For these reasons, several arenicin-1 variants with increased or modified symmetry, as well as with changes in other primary structural features, have been designed (Figure 1), chemically synthesized, and investigated. Molecular dynamics simulations allowed us to predict possible effects on the structures of these variants and to analyze their conformational stability and dynamics. A linearized version of arenicin-1, as well as two variants with β-strand permutations, ARin-s and ARs-C, showed a rapid and robust folding into a well-defined β-hairpin structure that showed a remarkable similarity with the experimentally determined structure of the parent peptide arenicin-1. The RMSD was relatively low (1.6–1.9 Å, Figure 2b), and the hydrogen bonding pattern between the flanking β-strands was the same (Figure 3), so that the overall geometric parameters, twist and kink angles, were virtually indistinguishable from those of arenicin-1. These simulations suggested that permutations of whole β-strands, including palindromic symmetrization, did not significantly affect the characteristic β-hairpin structure of the scaffold, although switching the VYAYV and RRYRVL motifs between N- and C-terminal strands (as in ARs-in), might affect the mechanism of self-association in a membrane environment, which is reported to involve the N-terminal strand of the native peptide.

On the other hand, MD simulations on peptides with a non-canonical, branched, parallel β-hairpin arrangement achieved by chemical synthesis supported our hypothesis that an increased symmetry in the primary structure would translate into a more symmetrical β-structure, with reduced twist and kink angles. For the two highly symmetric analogues of this type (ARs-N-B and ARs-C-B), the β-hairpin became almost flat, and the hydrogen bonding pattern between the strands was altered (Figure 3). This would result in a different pattern of vacant hydrogen donor/acceptors on the other side of the peptide’s β-strands, also affecting intermolecular contacts. Considering that native arenicin molecules form dimers by a parallel association of the N-terminal β-strands [5,11], the branched variants could have an altered oligomerization propensity and, therefore, a modified pore-forming activity. Their biological activity might be reduced.

In effect, the structure–antimicrobial activity relationship of arenicin-1 turned out to be rather robust, with all studied variants, except ARin-s (with an inverted strand arrangement) and the symmetrical variant ARs-N (with reduced charge), maintaining an appreciable activity against both Gram-negative and Gram-positive bacteria, including drug-resistant strains. The charge dependence of the activity for ARs-N (+5) was also suggested by the fact that the activity was partly re-established for the parallel-stranded analog ARs-N-B (+7), which is supported by similar observations for other AMPs [26,27]. The activity loss in ARin-s is significant, and as the secondary structure was modelled to be very similar to that of the parent peptide, could be due either to the altered residue arrangement or to a reduced propensity of the altered N-terminal strand to dimerize in the membrane.

The cytotoxic action of arenicin-1 variants towards mammalian cells was less sensitive to the molecule’s charge and more sensitive to its symmetry and three-dimensional organization. The flatter, symmetrically branched peptides ARs-N-B and ARs-C-B were less toxic to K-562 cells by a factor of two than the native peptide, whereas ARs-N and ARs-C—with a more pseudo-symmetric but antiparallel β-hairpin conformation—showed a comparable or slightly increased cytotoxicity. When native arenicin-1 dimerizes by the parallel association of its N-terminal strands, it adopts a more planar conformation [5,11]. The intrinsically more planar conformations of ARs-N-B and ARs-C-B might thus be expected to favor oligomerization, and in line with several studies, an increased propensity for self-association in solution should correlate with increased cytotoxicity [23,28,29]. The fact that these analogs showed both a reduced bacterial membrane permeability (Figure 5) and host cell cytotoxicity, compared to the parent peptide, suggests that this was not the case. Possibly, the different intramolecular H-bonding patterns of the parallel branches in the modified peptides affected their capacity to form the intermolecular H-bonds.

Analysis of other variants revealed that an improvement of the in vitro antimicrobial activity could be obtained by Arg-to-Lys (AR-K) or Trp-to-Phe (AR-F) substitutions, but at the expense of an even more prominently increased toxicity towards mammalian cells. Interestingly, other reports on Arg-to-Lys substitutions in AMPs [30,31] suggest that the arginine side chain can form more hydrogen bonds and thus interact more strongly with phospholipid components of bacterial membranes, with respect to lysine, which should enhance the activity of Arg-rich AMPs with respect to the Lys-rich analogues [31,32]. Previously published data on an arenicin-1 analogue with Arg-to-Lys substitutions indicated that the activity of this peptide is comparable to that of the parent AR, but in a salt-, medium- and bacterial species-dependent manner [7].

The replacement of Trp with Phe may favor the insertion of the AR-F peptide into membranes but it does not seem to discriminate between bacterial and eukaryotic membranes. Furthermore, it is reported that the kinked arenicin-1 hairpin becomes significantly flatter upon membrane contact and oligomerization [5]. Molecular dynamics simulations with ARs-C-B indicated that Trp stacking in parallel strands may contribute to stabilizing the flattened conformation.

The backbone-cyclized analogue of arenicin-1 acted similarly to the parent peptide towards both bacterial and mammalian cells, while the linear variant showed a higher selectivity for the bacterial cells. The macro-cyclization of AMPs has been reported to reduce the toxicity of some AMPs towards host cells (e.g., protegrin 1, tachyplesin 1, and gomesin AMPs [33,34,35]), but in the case of arenicin, it did not substantially alter the biological activity. In fact, as it occurs close to the disulphide bridge, it results in a rather tight, five-residue cycle (CWRWC), which could be quite rigid considering the steric bulk of the central residues. MD simulations with the linear AR variant showed that it has a strong propensity to fold to a β-hairpin even in the absence of a disulphide bridge, which suggests that backbone cyclization could replace disulphide bridging to stabilize the peptide, freeing the positions of the Cys residues for variations that may improve its activity. This has been observed for macrocyclic antimicrobial conotoxin analogues with replaced cysteines [36].

With respect to the linearized arenicin, a variant with the replacement of cysteine residues with serine has previously been reported and was shown to have a 2–4-fold decreased antimicrobial activity [3,7]. Our linearized analogue, with alkylated cysteine residues, instead substantially maintained the antimicrobial activity. As MD simulations suggested, it also displays a substantial propensity to fold to a β-hairpin conformation, albeit with a reduced stability with respect to the parent peptide; it may also maintain some of its capacity to oligomerize and permeabilize bacterial membranes. In any case, studies on other linearized β-hairpin AMPs with reduced Cys or Cys replaced with Ser (e.g., bovine cyclic dodecapeptide [37] and porcine protegrin 1 [38]) indicate that, while activity may be reduced to some extent, it is not entirely abrogated. In fact, some linearized analogues of bovine dodecapeptide (bactenecin 1) demonstrated a potent antibacterial action and higher selectivity for bacterial cells than the parent peptide [39]. Studies with cyclic, linear, or dimeric (parallel and antiparallel) cyclic dodecapeptide suggest that the antimicrobial activity is quite robust and retained to some extent by all these forms, even though characteristics such as the salt-dependence of the activity can vary [40].

## 4. Materials and Methods

### 4.1. Peptide Synthesis

#### 4.1.1. Antiparallel β-Hairpins

Solid-phase peptide synthesis of most variants was performed on a microwave-enhanced CEM Liberty synthesizer (Charlotte, NC, USA), loaded with Trp-substituted, 2-chlorotrityl chloride resin (substitution of 0.2 mmol/g). Insertion of the C-terminal Trp was performed manually according to the instructions provided in the synthesis notes section (section 2.17) of the Novabiochem catalogue. Double coupling was affected at all positions, with a four-fold excess of fluoren-9-dymethoxycarbonyl amino acid/HATU/diisopropylethylamine (1:1:1.7, by vol.) at 70 °C. For couplings involving Cys, the temperature was limited to 50 °C. Branched peptides (ARs-N-B and ARs-C-B) were synthesized as amides, using the NovaPEG Rink Amide resin (substitution 0.22 mmol/g). Diamminopropionic acid (Fmoc-DAP(Boc)-OH) was loaded as the C-terminal residue, followed by (Fmoc-Orn(Fmoc)-OH) and then branching from the Orn residue. Peptides were cleaved from the resin and deprotected using a cocktail consisting of trifluoroacetic acid (TFA), water, ethanedithiol, triisopropylsilane mixture (94:2.5:2.5:1, by vol.). The free peptides were precipitated and washed with t-butyl methyl ether and dried under nitrogen.

#### 4.1.2. Branched, Parallel Hairpins, and Simulation of the Turn Region

**Figure 7:**
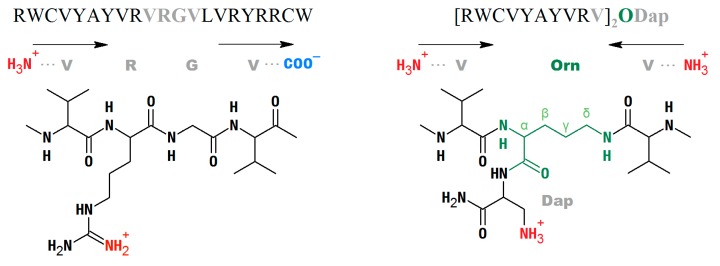
Sequence of the turn region of native arenicin (–VRGV–) and simulated turn region of the “branched” arenicin 1 variants (–VOrn(δ)V–) where the Orn α-amine is coupled to amidated Dap.

While the primary structure of the native peptide imposed an antiparallel arrangement of the strands in the β-hairpin [2], the synthetic variants with the parallel prongs of the hairpin were designed to achieve a more symmetric arrangement of the peptide molecule. In the design of these “branched” peptides, it was attempted to maintain a structure of the branching site as far as possible isosteric with the Arg residue normally present in the turn (Figure 7), by branching from an ornithine (Fmoc-Orn(Fmoc)-OH) coupled to a C-terminal Dap amide (Fmoc-Dap(Boc)-OH) coupled to a Rink amide resin]. The synthesis and cleavage were carried out in the same conditions as for the β-hairpin peptides. ARs-N-B and ARs-C-B have identical co-directional prongs in the two parallel strands of the branched peptide, based, respectively, on the N- or C- terminal strands of arenicin.

#### 4.1.3. Backbone-Cyclized Arenicin

The cyclic variant (ARcycl) was synthesized using the same Trp-substituted, 2-chlorotrityl chloride resin (substitution of 0.2 mmol/g), but with the sequence Boc–CVYAYVRVRGVLVRYRRCW, to provide an N-terminal Cys residue for native chemical ligation. The methodology has been described previously for native ligation of two peptide fragments [41] and was adapted for backbone cyclization. Briefly, the fully protected peptide carboxylic acid (including Boc-protected N-terminus) was cleaved from the resin with hexafluoroisopropanol (HFIP) in dichloromethane (DCM), and the product was esterified to the corresponding 4-acetamidophenyl-thioester, according to a published procedure [42,43]. After acid deprotection of this peptide thioaryl ester with a thiol free cleavage mixture, it was suspended to a concentration of 30 mM in a solution of 6 M guanidinium hydrochloride, 100 mM sodium acetate, 2 mM EDTA in the presence of 10 eq. of tris(2-carboxyethyl)phosphine (TCEP) to ensure the Cys residues were reduced, and left for 1 h. The backbone-cyclized peptide was then purified by RP-HPLC and resuspended in folding buffer (pH 8.5), and the disulphide bridge was formed as described below.

#### 4.1.4. Disulphide Bond Formation

Disulphide bonds in the arenicin variants were formed by adding the peptides to a freshly prepared N_2_-saturated aqueous buffer (1 M guanidinium chloride/0.1 M ammonium acetate/2 mM EDTA, pH 8.5) in the presence of 1 mM cysteine and 0.1 mM cystine. The final peptide concentration was kept below 10 µM to avoid dimer formation, and oxidation was carried out overnight. The linear variant of arenicin-1 was obtained by means of reduction of the parent AR followed by alkylation of the reduced peptide with iodacetamide using a standard protocol.

Folding was monitored by analytical RP-HPLC (GE Life Sciences Äkta FPLC 900 Pittsburg, PA, USA) using a Waters Symmetry® C18 column (3.5 µm, 100 Å, 4.6 mm × 50 mm), and after completion, the final desalting and purification were carried out using a Waters Delta-Pak® C18 column (15 µm, 300 Å, 25 mm × 100 mm). Gradients were typically 5%–55% acetonitrile in H_2_O (0.05% trifluoroacetic acid) for 30 min. The correct structure and purity were confirmed by ESI–MS using a Bruker Daltonics Esquire 4000 mass spectrometer (Billerica, MA, USA), working in positive mode, directly on the eluate fractions.

### 4.2. Molecular Modeling

#### 4.2.1. Molecular Dynamics Simulations

All MD simulations were performed with the *Gromacs-4.5.4* software package [44] with *GROMOS 43a2* forcefield [45]. Two types of initial conformations were used to simulate the folding and dynamics of the peptides: an extended linear conformation and a β-sheet structure. The preparation of the initial conformations is described in Supplementary materials. The topologies for ornithine and diaminopropionic acid in ARs-N-B and ARs-C-B were constructed manually, analogously to a lysine residue. The linearized version of arenicin-1 (ARlin) had reduced, carboxamidomethylated cysteines (RCM-Cys), and the cyclic variant (ARcycl) had an additional peptide bond between the terminal residues. All calculations utilized periodic boundary conditions. Long-range electrostatic interactions were computed by the particle-mesh Ewald (PME) method. The cut-off for non-bonded van der Waals interactions was set to 1.2 nm. Each system was first energy-minimized by 5000 steps of steepest descent. Energy minimization was followed by two 100 ps equilibration runs using NVT and NPT ensembles during which the positions of peptide atoms were restrained by a harmonic potential with a force constant of 1000 kJ∙mol^−1^∙nm^−2^. The equilibration was followed by 50–120 ns production MD runs integrated with a 2 fs timestep. During the MD simulations, temperature was maintained at 300–315 K by a Nose–Hoover thermostat with a time coupling constant 0.5 ps, and pressure was maintained at 1 atm by a Parrinello–Rahman barostat with a time coupling constant 2.0 ps. In the case of the peptides ARs-N-B and ARs-C-B, we also performed a periodic simulated annealing with temperatures at 300–365 K. The coordinates were written to an output trajectory file every 2 ps for further analysis. The resulting MD trajectories and the corresponding simulation parameters are summarized in Table S1.

#### 4.2.2. Analysis of the Simulation Results

The analysis of the MD trajectories was done using various utilities in the *Gromacs* package. To monitor the folding process, we calculated the RMSD of C^α^ carbon atoms from their initial positions. The evolution of the secondary structure was followed using the *DSSP* structure assignment program [46]. The stability and dynamics of the peptides were accessed by calculating RMSF of C^α^ carbon atoms and the average number of hydrogen bonds at the tail of a MD trajectory, where the system reached equilibrium. The same equilibrium parts of the trajectories were used to clusterize the conformations and determine the mean peptide structures. These mean structures were used to estimate the structural similarity with arenicin-1 by calculating the RMSD with experimental NMR structure PDB: 2JSB and to determine the secondary structure content by the STRIDE algorithm [47]. The kink and twist angles were analyzed analogously to a previous work on molecular dynamics simulation of arenicin-2 [6]; the details of the analysis methodology are given in Supplementary materials (Supplementary Chapter S1, [4,48,49,50]). The figures of the peptides were prepared using PyMol [49].

### 4.3. Antibacterial Assays

#### 4.3.1. Bacterial Strains

*E. coli* ML35p, *Listeria monocytogenes* EGD, methicillin-resistant *Staphylococcus aureus* (MRSA) ATCC 33591 were kindly provided by Prof. Robert Lehrer (University of California, Los Angeles, CA, USA); *E. coli* ATCC 25922, *E. coli* M15, *Pseudomonas aeruginosa* ATCC 27853, *S. aureus* ATCC 25923 were provided by Dr. Elena Ermolenko (Institute of Experimental Medicine, St-Petersburg, Russia); drug-resistant clinical isolates were provided by Prof. Gennadiy Afinogenov (Saint Petersburg State University, Russia); *S. aureus* 710A has been described previously [51]. The following clinical isolates of bacteria were used: *P.s aeruginosa* resistant to aztreonam, ceftazidime, cefotaxime (obtained from the urine of a patient with cystitis), *Acinetobacter baumannii* resistant to meropenem (from an infected wound); *Staphylococcus intermedius* (from an infected wound caused by a dog bite) resistant to ciprofloxacin, cefuroxime, clindamycin, erythromycin, rifampin, gentamicin, benzylpenicillin, oxacillin.

#### 4.3.2. Broth Microdilution Assay

This assay was applied to determine the MIC, according to the guidelines of the Clinical and Laboratory Standards Institute, using Mueller Hinton (MH) Broth. We prepared 2x stocks of peptides and serially diluted them in sterile PBS instead of Mueller Hinton Broth, so that the treatment of the bacteria with the peptides was carried out in a medium containing 50% of MH and 50% of PBS, as described [52]. The overnight cultures of each strain were transferred to fresh MH media and further incubated to obtain a mid-logarithmic-growth-phase culture of bacteria. The absorbance of each bacterial suspension was measured at 620 nm, and bacteria were then diluted to approximately 2 × 10^5^ CFU/mL. Then, 50 µL of the suspensions were mixed with 50 µL of the peptide dilutions in the wells of a microtiter plate (pre-treated for 1 h at 37 °C with 0.2% bovine serum albumin (BSA) in water, sterilized by filtration, to diminish the non-specific binding of the peptides to the plastic surfaces).

After incubation for 18 h at 37 °C, the MIC was read as the lowest concentration of antimicrobial agent resulting in the complete inhibition of visible growth; results were obtained from 3–5 independent determinations and are shown as medians.

The overall activity against a set of tested bacterial strains was determined by way of the commonly used geometric mean of measured MIC values [29,53,54]. Unlike the arithmetic mean, this parameter is less sensitive to positive outbursts (extreme values) [55], so that possible isolated cases of bacterial resistance do not dramatically outweigh all other MIC values. At the same time, the geometric mean can still discriminate antimicrobial agents showing minor variations in activity; in other words, it has a better “resolution” than the median MIC (MIC_50_) [54]. Some mathematical basics for choosing the geometric mean for antimicrobial activity comparison are further discussed in the Supplementary Chapter S3.

#### 4.3.3. Membrane Permeabilization Assay

The *E. coli* ML35p outer membrane permeability was assessed by monitoring hydrolysis of the chromogenic β-lactamase substrate nitrocefin (3-(2,4-dinitrostyryl)-(6R,7R)-7-(2-thienyl acetamido)ceph-3-em-4-carboxylic acid) (Calbiochem-Novabiochem, San-Diego, CA, USA) by detection of the hydrolysis product at 486 nm. Inner membrane permeability was monitored by measuring the hydrolysis of *o*-nitrophenyl-β-d-galactoside (ONPG, Sigma, La Jolla, CA, USA) at 420 nm [15]. The *E. coli* ML35p strain expresses a plasmid-encoded periplasmic β-lactamase; it constitutively expresses cytoplasmic β-galactosidase and lacks lactose permease [15]. *E. coli* ML35p was maintained on trypticase soy agar plates containing 100 mg of ampicillin per mL. The bacteria used for antimicrobial testing or membrane permeability assays were picked from a single colony, incubated in 50 mL of sterile Trypticase soy broth for 16 h at 37 °C, washed three times with 10 mM sodium phosphate buffer (pH 7.4), adjusted to an optical density at 620 nm of 1 (2.5 × 10^8^ CFU/mL), and kept on ice until use. The assays were performed in 96-well microtiter plates that were monitored every minute with a SpectraMax 250 Microplate Spectrophotometer (Molecular Devices, Sunnyvale, CA, USA) using the SOFTmax PRO software supplied by the manufacturer. The final incubation medium contained 10 mM sodium phosphate buffer, 100 mM NaCl, pH 7.4. Incubation wells (final volume of 100 μL) also contained either 2.5 mM of ONPG or 20 μM of nitrocefin, 2.5 × 10^7^ CFU/mL of washed, stationary-phase *E. coli* ML35p cells, and the peptide of interest at a concentration equal to 2× MIC or an equivalent volume of acidified water (negative controls). Assays were run at 37 °C, with 5 s of shaking every minute. The reactions were started by adding the bacteria. The data were processed using the Sigma Plot 11 software; the results of a typical experiment are presented at the Figure 5.

### 4.4. Cytotoxicity Assays

#### 4.4.1. Hemolytic Activity

Hemolysis assays were carried out on human red blood cells according to the ethical principles of the Declaration of Helsinki. Peripheral blood was drawn from healthy donors (written informed consent was obtained from all volunteers) into vacutainers containing EDTA under aseptic conditions and washed twice with an ice-cold phosphate buffered saline (PBS). The supernatant was discarded, and the pellet was resuspended in PBS. Hemolytic action was tested by incubating increasing concentrations of peptides with a suspension (2.5% v/v) of washed red blood cells in PBS. After 30 min at 37 °C, the tubes were centrifuged for 3 min at 10,000 g. Hemoglobin release was monitored at 540 nm using the SpectraMax 250 Microplate Spectrophotometer (Molecular Devices, Sunnyvale, CA, USA). Total lysis (100% hemolysis) was determined by adding 1% (v/v) Triton X-100, and the negative control value was determined by incubating red blood cells in buffer only. The percentage of hemolysis was calculated as:

% Hemolysis = ((*A*_exper_ − *A*_control_)/(*A*_total_ − *A*_control_)) × 100, (1)

where *A*_exper_ and *A*_control_ signify the absorbance values of the supernatants from treated and untreated red blood cells, and *A*_total_ is the supernatant of the cells treated with 1% Triton X-100. All evaluations were repeated in 3–6 separate experiments, carried out in triplicates.

#### 4.4.2. MTT Test

The standard MTT ((3-(4,5-Dimethylthiazol-2-yl)-2,5-diphenyltetrazolium bromide) test was used for the examination of the cytotoxic activity of AMPs [56] towards human erithroleukemia cells K-562. The cells were purchased from Biolot (Saint Petersburg, Russia) and were grown in RPMI 1640 medium (Biolot, Saint Petersburg, Russia) supplemented with glutamine and 10% FCS. Before the experiment, the culturing medium was replaced with serum-free RPMI 1640. Serial dilutions of the peptides in RPMI were plated in sterile 96-well microplates, and the target cells were dispensed to the microplates (10^5^ cells/well in RPMI 1640). The plates were incubated for 24 h at 37 °C under 5% CO_2_. Cell-free media and cells incubated without peptides served as controls. Four hours before the incubation ended, MTT in PBS (5 mg/mL) was added to each well. After the incubation was stopped by adding isopropanol/ 0.04 M HCl, the optical density was measured at 540 nm, subtracting the background absorbance at 690 nm. Toxicity was determined by nonlinear regression analysis of the corresponding dose–response curves using the Sigma Plot 11 program to calculate the IC_50_ values (the concentration of the test substance that reduced the OD_540_ capacity by 50%).

## 5. Conclusions

Antibiotic substances from marine invertebrates, and in particular antimicrobial peptides, are a promising source of novel anti-infective drugs. Among these, arenicin-1 isolated from the lugworm *A. marina* coelomocytes, is one of the most potent. It exerts a marked microbicidal activity towards a variety of Gram-negative and Gram-positive bacteria (including drug-resistant clinical isolates) and also possesses significant toxicity towards mammalian cells. An intriguing feature of this peptide is a markedly symmetric arrangement of some residues in its sequence. Several variants have been elaborated to elucidate the role of this sequence symmetry in the peptides’ function and mode of action, to determine the effect of structural variations such as backbone cyclization or linearization, and to probe the importance of key residues for microbicidal efficacy, membranolytic activity, and toxicity towards mammalian cells.

The results of this work allow us to make the following considerations:

- Stand inversion or palyndromic symmetrization of the arenicin scaffold does not greatly affect its twisted and kinked antiparallel β-sheet conformation, whereas symmetrization by artificially branching strands results in a flattened and more regular parallel β-hairpin;
- Inverting the strand residue arrangement of the native peptide causes a decrease in activity. This may be due to decreased capacity to oligomerize via the inverted N-terminal strand;
- A more symmetric, palindromic strand arrangement did not improve the activity and decreased it if accompanied by a reduced net charge;
- Increasing symmetry by artificially “branching” strands in a parallel hairpin arrangement allowed to recover the antimicrobial activity while reducing the cytotoxic activity;
- All variants with a modified symmetry demonstrated a reduced capacity to permeabilize the inner membrane of *E. coli* ML35, possibly pointing to a reduced capacity for oligomerization and/or pore formation.
- The backbone cyclization of the arenicin-1 molecule resulted in improved activity towards drug-resistant clinical isolates but did not markedly affect cytotoxicity.
- Linearization of the peptide somewhat increased selectivity, while not greatly altering antimicrobial activity.

These findings suggest that the residue layout of the arenicin-1 molecule plays a significant role in its biological activity, although the contribution of the peculiar, pseudo-symmetric arrangement of some residues is still unclear. Furthermore, several other characteristics of the peptides (charge, hydrophobicity, residues involved in oligomerization) must also be taken in account. We suggest that recent developments in the computational design of AMPs employing pattern discovery [57] and deep learning [58], trained also using data from this type of study, may allow for an efficient optimization of these entangled parameters. Our results indicate that this molecule from a marine animal can serve as a robust template for the elaboration of novel therapeutic agents and they add to a plethora of other studies showing that it is arduous to redesign synthetic antimicrobial peptides from natural ones, improving both efficacy and selectivity.

## Figures and Tables

**Table 1:** Structural analysis of the resulting peptide models.

Peptide	^(a)^ C^α^ RMSD with 2JSB (Å)	^(b)^ Average N° of H-Bonds (±SD)	^(c)^ Average 2^y^ Structure Content
AR	1.37	8.3 ± 1.2	14/21
ARin-s	1.67	8.3 ± 1.2	16/21
ARs-C	1.90	6.4 ± 1.1	14/20
ARs-N-B	4.29	7.1 ± 1.3	13/22
ARs-C-B	3.30	6.2 ± 1.1	10/20
ARlin	3.13	5.4 ± 1.2	9/21
ARcycl	1.57	7.1 ± 1.2	16/21

^(a)^ Root-mean-square deviation (RMSD) of the coordinates of C^α^ atoms in variant structures of the experimental arenicin-1 NMR structure. ^(b)^ Average number (N°) of mainchain H-bonds calculated from the equilibrium parts of the MD trajectories. ^(c)^ Ratio of residues in antiparallel or parallel β-sheet conformation assigned by the STRIDE algorithm to the total N° of residues. 2JSB: Protein Data Bank (PDB) ID of arenicin-1.

**Table 2:** Antimicrobial activity of the arenicin-1 variants (broth microdilution assay).

	Minimal Inhibitory Concentrations (MIC) ^a^, µM									
	AR	ARin-s	ARs-N	ARs-C	ARs-N-B	ARs-C-B	AR K	AR F	ARlin	ARcycl
**Gram-negative laboratory strains**										
*Escherichia coli* ML35p	1–2	4–8	16	1	4	1–2	1	1	1–2	1–2
*E. coli* ATCC 25922	2	8	>16	2	8	4	2	1	2	2
*E. coli* M15	2	8	>16	2	4	8	1	1	2	2
*Pseudomonas aeruginosa* ATCC 27853	2	4–8	>16	1	2	1	1–2	1	2	1
**Gram-positive laboratory strains**										
*Listeria monocytogenes* EGD	1–2	2	8	1	2–4	2	2	2	2	2
*Staphylococcus aureus* 710A	2	16	>16	2–4	8	4	2	2	2	2
*S. aureus* ATCC 25923	2	16	>16	4	8	4	2	2	2	2
*MRSA* ATCC 33591	4	>16	>16	4–8	8	8	2	2	4	4
**Clinical isolates**										
*P. aeruginosa c.i.*	4	>16	>16	16	16	16	2–4	2	4–8	2
*Acinetobacter baumanii c.i.*	4	16	>16	2	4	8–16	1	2	4	4
*Staphylococcus intermidius*	8	>16	>16	8	8	4	4	4	8	4
*S. aureus c.i.*	4	>16	>16	16	8	4–8	4	4	8–16	4
**Overall statistics**										
G-MIC ^b^	2.7	≥12.8	≥26.9	3.2	5.9	4.4	1.9	1.8	3.1	2.3
G-MIC improvement ratio in comparison with AR ^c^	1.0	≤0.2	≤0.1	0.8	0.5	0.6	1.4 *	1.5 *	0.9	1.2 *

^a^ Minimal inhibitory concentrations (MIC) values were derived from 3–5 experiments which were performed in triplicates. ^b^ Geometric mean of the MIC (G-MIC) is the geometric mean of all determined MICs; the median values of MICs were used for calculation. In the case of MIC > 16 µM, the next concentration in the series of two-fold dilutions (32 µM) was used for G-MIC assessment. ^c^ As the lower MIC corresponds to the higher activity, MIC improvement was calculated as a ratio of G-MIC of native arenicin (AR) to G-MIC of the peptide of interest. *: higher overall activity, compared to AR.

**Table 3:** Cytotoxic effects of arenicin-1 variants towards human erythroleukemia K-562 cells and human erythrocytes.

	Effects of the Peptides									
	AR	ARin-s	ARs-N	ARs-C	ARs-N-B	ARs-C-B	AR-K	AR-F	ARlin	ARcyclic
**Cytotoxicity towards K-562 cell line (human erythroleukemia cells)**										
IC_50_^a^, µM (MTT-assay)	17.9	37.7	18.0	11.6	35.1	39.0	7.7	9.3	>40	16.2
SI_1_ assessment IC_50_/G-MIC	6.6	≤2.9	≤0.7	3.6	6.0	8.8	4.0	5.2	>12.8	7.0
SI_1_ improvement ratio in comparison with AR^b^	1.0	≤0.4	≤0.1	0.5	0.9	**1.3** *	0.6	0.8	**>1.9** *	**1.1**
**Hemolysis of human red blood cells**										
HC_50_^c^, µM	66.3	>80	>80	66.0	>80	>80	63.0	60.5	>80	65.5
SI_2_ assessment HC_50_/G-MIC	24.6	-	-	20.6	>13.7	>18.1	33.1	34.0	>25.6	28.2
SI_2_ improvement ratio in comparison with AR^b^	1.0	-	-	0.8	**>0.6**	**>0.7**	**1.3**	**1.4**	**>1.0**	**1.1**
HC_15_^c^, µM	10.3	17.2	23.5	11.8	12.4	56.4	10.1	10.6	29.9	14.0
SI_3_ assessment HC_15_/G-MIC	3.8	≤1.3	≤0.9	3.7	2.1	12.7	5.3	6.0	9.5	6.0
SI_3_ improvement ratio in comparison with AR^b^	1.0	≤0.4	≤0.2	**1.0**	0.6	**3.3** *	**1.4**	**1.6**	**2.5** *	**1.6**

^a^ Half-maximal inhibitory concentration (IC_50_) was calculated using Sigma Plot Standard Curve Analysis based on 3–4 independent experiments. ^b^ Selectivity index (SI) improvement was calculated as a ratio of the SI of the peptide of interest to the SI of native AR. The peptides with the highest SI values are the least cytotoxic. ^c^ Half-maximal hemolytic concentration (HC_50_) and 15% maximal hemolytic concentration (HC_15_) were calculated using Sigma Plot Standard Curve Analysis based on 3–8 independent experiments. *: variants with the most significant reduction of toxicity in both hemolysis and MTT-test, compared to AR.

## Supplementary Materials

The following are available online at https://www.mdpi.com/1660-3397/17/6/376/s1, Chapter S1: Molecular dynamics simulation protocol; Chapter S2: Analysis of the simulation results; Chapter S3: Why use geometric mean of MIC as an assessment of overall antimicrobial activity?
