# Supplementary material for: Redesigning Arenicin-1, an Antimicrobial Peptide from the Marine Polychaeta Arenicola marina, by Strand Rearrangement or Branching, Substitution of Specific Residues, and Backbone Linearization or Cyclization
Source: Mar Drugs. 2019 Jun 23;17(6):376. doi: 10.3390/md17060376 (PMC6627698; doi:10.3390/md17060376)
Supplement: Supplementary file 1 [file marinedrugs-17-00376-s001.pdf]

marine drugs

MDPI

Supplementary material

Redesigning Arenicin-1, an Antimicrobial Peptide from the Marine Polychaeta *Arenicola marina*, by Strand Rearrangement or Branching, Substitution of Specific Residues, and Backbone Linerization or Cyclization

Dmitriy S. Orlov [1,#], Olga V. Shamova [1,#] *, Igor E. Eliseev [2], Maria S. Zharkova [1], Oleg B. Chakchir [2], Nikolinka Antcheva [3], Sotir Zachariev [4], Pavel V. Panteleev [5], Vladimir N. Kokryakov [1], Tatiana V. Ovchinnikova [5,6], Alessandro Tossi [3,]

[1] Institute for Experimental medicine, 12 Academic Pavlov str., St. Petersburg 197376, Russia; ds-orlov@yandex.ru (D.S.O.); oshamova@yandex.ru (O.V.S.), kokryak@yandex.ru (V.N.K.), manyvel@mail.ru (M.S.Z.)
[2] St. Petersburg Academic University, 8/3 Khlopina str., St. Petersburg 194021, Russia; eliseev@spbau.ru (I.E.E.); chakchir@spbau.ru (O.B.C.)
[3] University of Trieste, Department of Life Sciences, Building Q, Via Giorgieri 5, Trieste 34127, Italy; nantcheva@gmail.com (N.A.); atossi@units.it (A.T.)
[4] International Centre for Genetic Engineering and Biotechnology, AREA Science Park, Padriciano 99, Trieste 34149, Italy; sotir@icgeb.org (S.Z.)
[5] M.M. Shemyakin & Yu.A. Ovchinnikov Institute of Bioorganic Chemistry, the Russian Academy of Sciences, Mikhluho-Maklaya str. 16/10, Moscow 117997, Russia; alarm14@gmail.com (P.V.P.); ovch@ibch.ru (T.V.O.)
[6] Department of Biotechnology, I.M. Sechenov First Moscow State Medical University, Moscow 119991, Russia

[#] First and second authors contributed equally to this work

* Correspondence: oshamova@yandex.ru; Tel.: +7 911 2530929

Received: date; Accepted: date; Published: date

S1. Molecular dynamics simulation protocol

Obtaining a quantitative measure of sequence symmetry

To obtain a quantitative measure of symmetry, we inverted each sequence and calculated N_{ident}— the number of identical residues after aligning the original and inverted sequence using Needleman–Wunsch algorithm [48]. The quantitative measure of sequence symmetry S was defined as the ratio of identical residues for the original and inverted sequence to the total sequence length N:

$$S = N_{ident} / N \quad (1)$$

Preparing initial conformations

Two types of initial conformations were used to simulate the folding and dynamics of the peptides: an extended linear conformation, and a β-sheet structure. Linear conformations for all peptides were created manually in PyMol [49]. An experimental NMR structure (PDB: 2JSB) [4] was used to simulate the original arenicin-1 (AR) and its cyclic variant (ARcycl). Initial β-sheet structures for ARin-s and ARs-C were obtained by replica-exchange Monte Carlo simulation in the Quark

program [50]. For peptides ARs-N-B and ARs-C-B, having non-standard amino acids, β-sheet structures were constructed manually using PyMol. All peptides except AR were first simulated with both linear and β-sheet structures lacking a disulfide bond to ensure unobstructed sampling of the conformational space. In the case of ARs-C, the disulfide bond was introduced for the next computational experiment after the cysteine residues came in close contact (<4 Å). The peptide models were placed in a simulation box which was filled with SPC water molecules. The system was neutralized by the addition of sodium and chlorine ions to a final concentration of approximately 0.15 M.

Simulation parameters

The resulting MD trajectories and the corresponding simulation parameters are summarized in Table S1.

Table S1. Molecular dynamics simulation details.

Peptide	Trajectory	Length, ns	Initial conf.	S–S bond	T, K	Box size, nm	Final conf.
ARlin	lin-1	50	linear	None** (RCM-Cys)	315	4.06	β
	**lin-2***	50	β (after lin-1)	None** (RCM-Cys)	300	5.01	β
AR	ar1-1	50	β (2JSB)	yes	315	4.05	β
	**ar1-2***	50	β (2JSB)	yes	315	6.04	β
ARcycl	**cycl***	50	β (2JSB) with a peptide bond R_1–W_{21}	yes	315	4.07	β
ARin-s	ar2-lin	50	linear	no	315	7.08	β
	**ar2***	100	β (Quark model)	no	315	5.05	β
ARs-C	ar4-lin1	100	linear	no	315	6.04	β
	ar4-lin2	120	β (after ar4-lin1)	yes	315	5.03	β
	ar4-1	50	β (Quark model)	no	315	4.06	β
	**ar4-2***	50	β (after ar4-1)	yes	315	4.05	β
ARs-N-B	ar5-lin	100	linear	no	315	6.04	coil
	ar5-anneal	50	β (prepared manually)	yes	300–365	5.51	β
	**ar5***	50	β (after ar5-anneal)	yes	300	5.01	β
ARs-C-B	ar6-lin	100	linear	no	315	7.07	coil
	ar6-anneal	50	β (prepared manually)	yes	315–365	5.04	β
	**ar6***	50	β (ar6-anneal)	yes	300	5.01	β

* Trajectories marked with asterisk were used for a detailed analysis.

** Reduced cysteines, carboxamidomethylated using iodoacetamide

S2. Analysis of the simulation results

Calculation of the kink and twist angles

The twist was defined as the angle between two vectors ***a*** and ***b***, where ***a*** pointed from the backbone C atom of the second amino acid (W^2 in AR) to the backbone N atom of the last amino acid (W^{21} in AR), and ***b*** pointed from the backbone C atom of the last amino acid before the turn (V^{10} in AR) to the backbone N atom of the first amino acid after the turn (V^{13} in AR). To analyze the kink angle, we chose the residues in the center of each β-strand, i.e. the 6th and 17th amino acids in the case of arenicin-1. The kink of the first strand was defined as the angle between a vector $\boldsymbol{c_1}$, pointing from the backbone C atom of the center residue to the C atom of the first residue in a strand (W^2 in AR), and a vector $\boldsymbol{d_1}$, pointing from the backbone C atom of the center residue to the C atom of the last residue in a strand (V^{10} in AR). The same calculation was made for the second strand, and the overall kink was estimated as the mean of the two angles.

Monitoring peptide folding

The process of the folding was monitored by determining the root-mean-square deviation (RMSD) from the initial linear structure (a) and the formation of secondary structure elements (b). Data for the rapid folding of the peptide ARs-C is presented in Figure S1a and S1b, respectively.

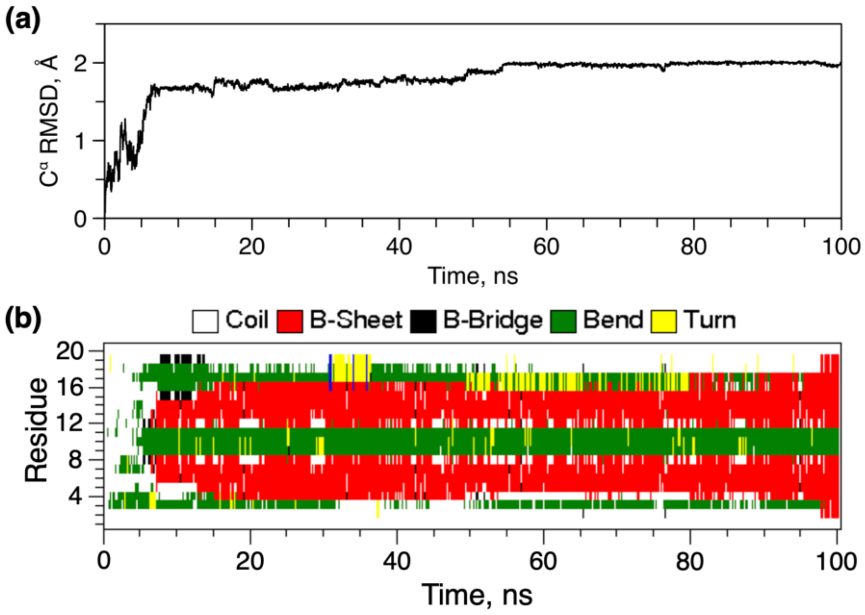

Figure S1. (**a**) Folding of the peptide ARs-C, monitored by a distance (RMSD) from initial linear conformation; (**b)** formation of secondary structure elements during the folding of the peptide ARs-C, monitored by *DSSP* program.

Analysis of peptide dynamics and stability

The dynamics of the peptide backbone were assessed by calculating root-mean-square fluctuations (RMSF) the C^{α} atoms. The characteristic for β-hairpin peptides graphs with labile N-, C-termini and turn region are given in Figure S2a. The stacking of N-terminal tryptophan residues, which contributes to higher rigidity of the peptide ARs-C-B, is shown in Figure S2b.

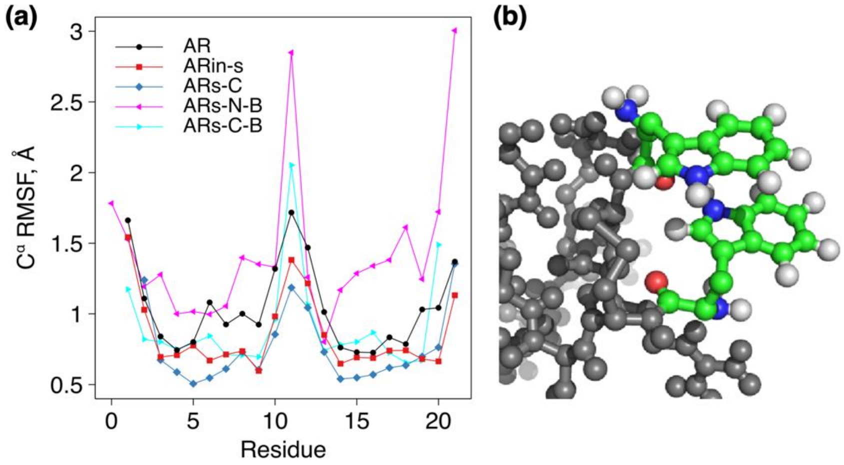

Figure S2. (a) Fluctuations of the positions of the Cα atoms (RMSF) show a typical pattern for β-hairpin peptides; **(b) stacking of N-terminal Trp residues** in ARs-C-B confers additional rigidity to the peptide structure.

S3. Why use geometric mean of MIC as an assessment of overall antimicrobial activity?

There are different measures and approaches to assess so called "general tendency" of obtained data, in other words to represent a dataset by a single value. The most common measures for this are the mean, which can be arithmetical, geometric, harmonic, etc., the median and the mode [55]; other methods, e.g. weighted sums, can also be involved. Which one to use in any particular case depends on the problem that needs to be solved, and on the question(s) that need to be answered. So, why use the geometric mean of minimal inhibitory concentrations (MIC) to compare the antimicrobial activities of substances?

A first step is to formulate the problem. Given two substances, A and B, with activities A_A and A_B, respectively, we need a way to quantitatively compare these activities, so that in the end we can state "the activity of A is higher than that of B by a factor of *f* ". This can be represented as:

$$A_A = f \cdot A_B. \quad \text{(s1)}$$

If we test the efficacy of substances A and B against only one bacterial strain, we just compare the commonly used measure of antimicrobial activity, the MIC value. The lower the MIC, the higher is the activity, so potency is inversely proportional, and if the MIC of substance A [MIC(A)] is 2 μM and MIC of substance B [MIC(B)] is 8 μM, then the antimicrobial activity of A is higher than the activity of B by a factor of 4, and Equation s1 becomes:

$$A_A = \frac{\text{MIC(B)}}{\text{MIC(A)}} \cdot A_B \quad \Rightarrow \quad f = \frac{\text{MIC(B)}}{\text{MIC(A)}}. \quad \text{(s2)}$$

How should one combine individual *f* values when the activity of substances A and B are assayed against several different bacteria? Should we sum them, or intermultiply, or, use some more complex treatment?

Let's consider an exemplary situation. Substances A and B were tested against two bacteria. Substance A was 4 times more effective than substance B against bacteria 1 [e.g. $\text{MIC(A)}_1 = 2$ μM, $\text{MIC(B)}_1 = 8$ μM, so that $f_1 = 4$], but substance B was 4 times more effective than A against bacteria 2 [e.g. $\text{MIC(A)}_2 = 8$ μM, $\text{MIC(B)}_2 = 2$ μM, so $f_2 = ¼$]. Putting aside any additional considerations about which bacterium is more desirable to eliminate, we can say that overall, the activities of A and B are equal. Mathematically speaking, we anticipate that overall $A_A = A_B$ so that according to Formula s1, $f = 1$. The easiest way to obtain a value of 1 by operating with $f_1 = 4$ and $f_2 = ¼$ is to intermultiply them. So a first model for calculating the overall *f* (f_{total}) in case of several tested bacteria is as the product of multiplication of *f*-s for individual bacteria:

$$f_{total} = \prod_{i=1}^{n} f_i = \prod_{i=1}^{n} \frac{\text{MIC(B)}_i}{\text{MIC(A)}_i}, \tag{s3}$$

where i is the index of an individual bacterial strain, f_i is factor f, calculated for bacterial strain i according to Formula s2, and n is the total number of tested strains.

We can test Equation s3 in another exemplary situation. Substances A and B were tested against 3 bacteria, and in each case A was found to be 4 times more active than B: $f_1 = f_2 = f_3 = 4$. Common sense leads one anticipates that the overall activity of A is higher than the overall activity of B by a factor of 4. However, according to the Equation s3 $f_{total} = f_1 \cdot f_2 \cdot f_3 = 4^3 = 64$. Moreover, if we test A and B against a 4th bacterium with the same result of $f_4 = 4$, f_{total} will become 4^4, and in case of n bacteria tested with equal results it will be $f_{total} = 4^n$. This does not make sense so our model clearly requires optimization. For instance, we could calculate some average value for f and so remove the incremental dependency on the number of bacterial species tested (n). It will additionally allow us to compare activities of substances tested on a different number of different bacterial species, at least to some extent.

An obvious method to remove the n power from 4^n in the current simplified example is to calculate its n-th root: $(4^n)^{1/n} = 4$, which was the anticipated answer. In general, the f values can be different for each bacterial strain (e.g. $f_1 \neq f_2 \neq \ldots \neq f_n$), and the upgraded equation for f_{total} will look like:

$$f_{total} = \left(\prod_{i=1}^{n} f_i\right)^{1/n} = \left(\prod_{i=1}^{n} \frac{\text{MIC(B)}_i}{\text{MIC(A)}_i}\right)^{1/n} = \frac{\left(\prod_{i=1}^{n} \text{MIC(B)}_i\right)^{1/n}}{\left(\prod_{i=1}^{n} \text{MIC(A)}_i\right)^{1/n}}. \tag{s4}$$

By definition, the geometric mean of a value (in this case G-MIC) is calculated as:

$$\text{G-MIC} = \left(\prod_{i=1}^{n} \text{MIC}_i\right)^{1/n}, \tag{s5}$$

where i indicates an individual bacterial strain, so MIC_i is MIC of the substance against bacterial strain i, and n is the total number of tested strains.

Thus, combining s1, s4 and s5, a final equation for comparing antimicrobial activities (A_A and A_B) of two substances A and B is:

$$A_A = \frac{\text{G-MIC(B)}}{\text{G-MIC(A)}} \cdot A_B\,; \qquad f_{total} = \frac{\text{G-MIC(B)}}{\text{G-MIC(A)}}. \tag{s6}$$

This retains the advantage of using a mean which takes into account that a different number of strains may be used with different substances, so the geometric mean is a reasonable choice to assess the general tendency in a set of MIC values, obtained by investigating the activity of substances against different bacteria, if these activities are to be compared.

© 2019 by the authors. Submitted for possible open access publication under the terms and conditions of the Creative Commons Attribution (CC BY) license (http://creativecommons.org/licenses/by/4.0/).
